# Impact of Sacubitril/Valsartan on Cardiac Autonomic Function Assessed Using Physiological Data from Implantable Cardioverter-Defibrillators

**DOI:** 10.3390/jcm15020719

**Published:** 2026-01-15

**Authors:** Lucy Barone, Domenico Sergi, Giampiero Maglia, Luca Bontempi, Marzia Giaccardi, Matteo Baroni, Claudia Amellone, Antonio Curnis, Giuliano D’Alterio, Davide Saporito, Paolo Vinciguerra, Simone Cipani, Patrizio Mazzone, Massimo Giammaria, Gianfranco Mitacchione, Daniele Masarone, Francesca Fabbri, Andrea Vannelli, Irene Baldassarre, Martina Del Maestro, Daniele Giacopelli, Eduardo Celentano, Gabriele Zanotto, Francesco Barillà

**Affiliations:** 1 Department of Systems Medicine, Policlinico Tor Vergata: Fondazione PTV, 00133 Rome, Italy; 2 Tirrenia Hospital, 87021 Belvedere Marittimo, Italy; 3 Department of Cardiology, Bolognini Hospital, 24068 Seriate, Italy; 4 Department of Cardiology, Santa Maria Annunziata Hospital, 50012 Florence, Italy; 5 Pediatric Cardiology Unit, Meyer Children’s Hospital IRCCS, 50139 Florence, Italy; 6 De Gasperis Cardio Center, Electrophysiology Unit, Niguarda Hospital, 20162 Milan, Italy; 7 Cardiology Department, Maria Vittoria Hospital, 10144 Turin, Italy; 8 Department of Electrophysiology and Cardiac Pacing, ASST Spedali Civili, 25121 Brescia, Italy; 9 Department of Medical and Surgical Specialties, Radiological Sciences and Public Health, University of Brescia, 25121 Brescia, Italy; 10 Cardiology Department, Monaldi Hospital, 80131 Naples, Italy; 11 Cardiology, Infermi Hospital, 47923 Rimini, Italy; 12 Clinical Research Unit, BIOTRONIK Italia, 20093 Cologno Monzese, Italy; 13 Department of Electrophysiology, Humanitas Gavazzeni, 24125 Bergamo, Italy; 14 Azienda Ulss 9 Scaligera, 37069 Villafranca, Italy; 15 Health Sciences, UniCamillus—Saint Camillus International University, 00131 Rome, Italy

**Keywords:** sacubitril/valsartan, heart failure, implantable cardioverter-defibrillator, remote monitoring, electrical parameters

## Abstract

**Background/Objectives**: Sacubitril/Valsartan is a cornerstone therapy to improve outcomes in patients with heart failure with reduced ejection fraction (HFrEF). This study aimed to investigate the effect of Sacubitril/Valsartan on cardiac autonomic balance using physiological sensor data obtained from implantable cardioverter-defibrillators (ICDs) or cardiac resynchronization therapy defibrillators (CRT-Ds). **Methods**: This observational study involved 54 ICD and CRT-D patients who initiated Sacubitril/Valsartan therapy to treat HFrEF. The evaluated key parameters included heart rate variability (HRV), 24 h mean heart rate (24 h-HR), and nocturnal heart rate (nHR). Device electrical parameters and ventricular arrhythmias were also assessed. The data were collected by remote monitoring and averaged over a 7-day window at baseline (before treatment) and at 3 and 12 months after treatment initiation. **Results**: Sacubitril/Valsartan significantly improved HRV at 3 months (from 78.6 ms [interquartile range: 54.2–104.6] to 80.8 ms [60.8–108.0]; *p* = 0.041), reduced 24 h-HR (from 73.2 bpm [67.3–77.7] to 69.9 bpm [64.2–75.7]; *p* = 0.016), and reduced nHR (from 63.0 bpm [58.1–70.0] to 60.4 bpm [56.0–68.6]; *p* = 0.028). No significant changes in HRV, 24 h-HR, and nHR were observed between 3- and 12-month follow-up. The device electrical parameters were not influenced by the treatment. While the overall ventricular arrhythmia burden did not change post-treatment, patients with pre-treatment arrhythmias experienced a significant reduction in episodes from 2.97 (pre-treatment) to 0.82 (post-treatment) events per 100 patient years (*p* = 0.008). **Conclusions**: Sacubitril/Valsartan therapy in HFrEF patients was associated with statistically significant changes in cardiac autonomic indices, including a small increase in HRV and a slight reduction in heart rate, mainly during the first three months of treatment.

## 1. Introduction

Heart failure (HF) remains a leading cause of morbidity, mortality, and hospitalization worldwide [1]. In patients with HF with reduced ejection fraction (HFrEF), treatment involves both pharmacological and non-pharmacological approaches. Among the pharmacological options, the combination of Sacubitril, a neprilysin inhibitor, and Valsartan, an angiotensin II receptor blocker, is considered a cornerstone therapy. The PARADIGM-HF trial demonstrated the efficacy of Sacubitril/Valsartan in significantly reducing cardiovascular and all-cause mortality compared to Enalapril, an angiotensin-converting enzyme inhibitor [2]. Recent studies further suggest that Sacubitril/Valsartan reduces the incidence of ventricular arrhythmias and the frequency of appropriate defibrillator shocks, though the underlying mechanisms remain unclear [3,4,5]. In patients with HF, autonomic dysfunction is characterized by an imbalance between heightened sympathetic activity and diminished parasympathetic activity [6,7]. Chronic sympathetic overactivation leads to β_1_-adrenergic receptor downregulation, adverse cardiac remodeling, and an increased risk of life-threatening ventricular arrhythmias. These factors contribute to the elevated risk of sudden cardiac death observed in HFrEF patients [8].

Heart rate variability (HRV) analysis is a common method for assessing cardiac autonomic nervous system activity. Reduced HRV has been linked to a higher risk of arrhythmic events in HF patients [9,10,11]. Some studies have suggested that Sacubitril/Valsartan may favorably modulate cardiac autonomic function, potentially enhancing parasympathetic activity, though these effects are still considered mechanistic hypotheses rather than definitively proven clinical outcomes [12]. This study aimed to further elucidate the effects of Sacubitril/Valsartan on cardiac autonomic modulation in HFrEF patients by analyzing physiological sensor data from continuous remote monitoring of implantable cardioverter-defibrillators (ICDs).

## 2. Materials and Methods

### 2.1. Study Design

This analysis pooled data from a single-center observational study and the multicenter observational Home Monitoring Expert Alliance (HMEA) project [13]. In total, the dataset reflects contributions from eight Italian centers, representing a multicenter experience. Data were prospectively collected through automatic remote monitoring of implantable devices during routine patient care. Both projects received approval from competent ethics committees, and all patients provided informed consent for data processing.

### 2.2. Study Objective and Population

The primary aim of this study was to evaluate the effect of initiating Sacubitril/Valsartan treatment on cardiac autonomic function in patients with chronic HF and reduced ejection fraction. This was assessed using physiological parameters related to heart rate, as transmitted daily by implantable devices. Additionally, potential effects on device electrical parameters and ventricular arrhythmic risk were investigated.

We included patients with HF of various etiologies and a left ventricular ejection fraction ≤35% who had an implanted ICD or cardiac resynchronization therapy defibrillator (CRT-D) equipped with automatic remote monitoring. Sacubitril/Valsartan treatment had to be initiated according to clinical practice, with remote transmissions available for at least one month before and one year after therapy initiation. Patients were excluded if they were in atrial fibrillation (AF) at the time of therapy initiation.

### 2.3. Data of Interest and Analysis

This study focused on the following heart rate related physiological parameters, which were measured and transmitted daily by the devices:

1. HRV, calculated as the standard deviation of the mean atrial interbeat interval of normal sinus beats in 5 min segments over 24 h [14].
2. Mean heart rate over 24 h (24 h-HR).
3. Nocturnal heart rate (nHR), defined as the lowest heart rate value during the resting period (1 a.m. to 5 a.m.).
4. Frequency of premature ventricular contractions per hour (PVC/h).
5. Percentage of cardiac resynchronization therapy pacing (CRT%).
6. Device-detected atrial fibrillation burden, calculated as the cumulative time spent in atrial arrhythmias over 24 h, based on a device-detected rate threshold of ≥200 beats per minute.
7. Physical activity, measured by an accelerometer sensor and reported as a percentage of 24 h.

Among the device electrical parameters, we analyzed the right ventricular (RV) lead to assess the following: (i) R-wave amplitude; (ii) RV pacing threshold; (iii) RV pacing impedance; (iv) shock impedance. All of these parameters were automatically measured and transmitted daily.

Physiological and electrical parameters were averaged over a 7-day window at three specific time points related to Sacubitril/Valsartan initiation: before treatment, at 3 months, and 12 months after treatment initiation. HRV could not be calculated in patients with single-chamber ICDs or in those experiencing AF during the analyzed period; therefore, HRV analyses were restricted to patients in sinus rhythm.

All sustained ventricular arrhythmias (VAs) recorded by the devices from the time of implantation to the last available remote transmission were collected. These events were adjudicated for appropriateness by an expert electrophysiologist who was blinded to the timing of occurrence.

### 2.4. Statistics

Descriptive statistics for continuous variables are presented as medians with interquartile ranges (IQRs), while categorical variables are expressed as percentages, calculated based on non-missing values. Differences between subgroups were analyzed using the Kruskal–Wallis rank test for continuous variables and the Pearson χ^2^ test for categorical variables, as appropriate. Changes in physiological and electrical parameters between consecutive time points were evaluated using linear mixed models. Fixed effects were assigned to time points, with random intercepts at the patient level. The Wilcoxon signed-rank test was used to compare event rates before and after Sacubitril/Valsartan treatment initiation. Statistical significance was defined as a *p*-value < 0.05. All analyses were performed using STATA/MP 18.0 (StataCorp, College Station, TX, USA).

## 3. Results

### 3.1. Sample Characteristics

The cohort consisted of 54 patients with HFrEF who were initiated on Sacubitril/Valsartan therapy during remote monitoring of their implanted devices. The median age of the cohort was 68 years (IQR: 63–73), and 85% were male. Implanted devices were manufactured by Biotronik SE & Co. KG (Berlin, Germany), including the Acticor, Rivacor, Ilivia, Intica, Itrevia, and Iperia models. Twenty-seven patients (50%) had a CRT-D, 15 patients (28%) had a dual-chamber ICD, 11 patients (20%) had a single-lead ICD with atrial sensing dipole (DX ICD, Biotronik SE & Co. KG, Berlin, Germany), and one patient (2%) had a conventional single-chamber ICD. The majority (75%) were classified as New York Heart Association (NYHA) functional class II. More than half (59%) presented with ischemic cardiomyopathy. Detailed patient characteristics are shown in Table 1. Sacubitril/Valsartan therapy was initiated a median 209 days (IQR: 180–643) after device implantation.

### 3.2. Physiological Parameters

Device-detected physiological parameters assessed at three specific time points related to Sacubitril/Valsartan initiation: pre-treatment (pT), at 3 months after therapy initiation (3 mT), and at 12 months (12 mT), are summarized in Table 2.

For HRV, valid data were available for 39 patients at baseline, 38 at 3 months, and 34 at 12 months after excluding patient with single-chamber ICD and patients with periods of AF.

From pT to 3 mT, Sacubitril/Valsartan therapy significantly:

- increased HRV (pT: 78.6 ms [54.2–104.6] vs. 3 mT: 80.8 ms [60.8–108.0]; *p* = 0.041);
- decreased 24 h-HR (pT: 73.2 bpm [67.3–77.7] vs. 3 mT: 69.9 bpm [64.2–75.7]; *p* = 0.016);
- decreased nHR (pT: 63.0 bpm [58.1–70.0] vs. 3 mT: 60.4 bpm [56.0–68.6]; *p* = 0.028).

**Figure 1:**
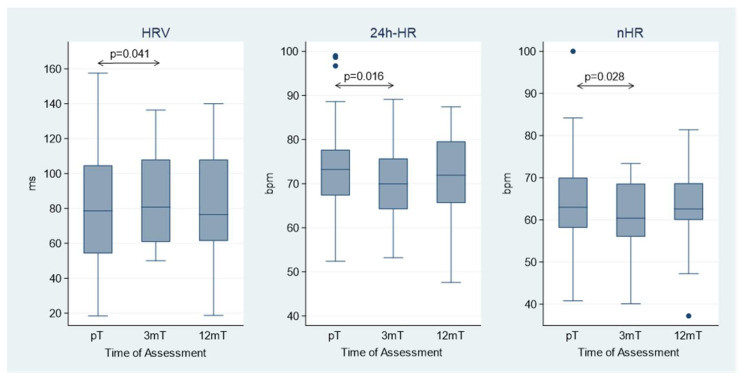
Heart rate variability (HRV), mean heart rate (24 h-HR), and nocturnal heart rate (nHR) at the three time points: before Sacubitril/Valsartan treatment (pT), at 3-month (3 mT), and 12-month (12 mT) follow-up after treatment initiation. The box represents the interquartile range (IQR) of the data, with the horizontal line inside the box indicating the median. The whiskers extend to the minimum and maximum values within 1.5 × IQR from the lower and upper quartiles, respectively. Data points outside this range are shown as individual points and represent outliers.

No significant changes in these parameters were observed between the 3-month and 12-month follow-up (Table 3). Histograms illustrating the variations in HRV, 24 h-HR, and nHR following Sacubitril/Valsartan initiation are presented in Figure 1.

Additionally, no significant variations were noted in PVC/h, CRT%, device-detected AF, or patient activity either between pre-treatment and 3-month follow-up or between pre-treatment and 12-month follow-up (Table 3).

### 3.3. Device Electrical Parameters

Device electrical parameters for each time point (pre-treatment, 3 months, 12 months) are summarized in Table 2. The analysis using mixed models revealed no significant differences across the three time points (Table 3).

### 3.4. Sustained Ventricular Arrhythmias

Over the median period of 209 days (IQR: 180–643) between device implantation and Sacubitril/Valsartan initiation, 15 patients (27.8%) had episodes of sustained VA. During a subsequent longer follow-up period after therapy onset (median 438 days [IQR: 365–1132]), a total of 17 patients (31.5%) had sustained VA, with a median time until the first episode of 353 days (IQR: 269–811) after therapy initiation. The overall rate of VA was not different between pre- and post-treatment (0.82 vs. 0.44 episodes per 100 patient years, respectively; *p* = 0.798). However, in a subgroup of patients with sustained VA before treatment, the event rate decreased significantly after therapy initiation—from 2.97 (pre-treatment) to 0.82 (post-treatment) episodes per 100 patient years (*p* = 0.008).

## 4. Discussion

This study provides insights into the impact of Sacubitril/Valsartan therapy on cardiac autonomic modulation in patients with HFrEF, utilizing physiological sensor data from continuous remote monitoring of ICD and CRT-D devices. Our findings highlight three key observations:

- Improvement in HRV and HR: Within 3 months of initiating Sacubitril/Valsartan therapy, we observed a small improvement in HRV and a modest reduction in 24 h-HR and nHR, despite the limited statistical power due to small sample size. These changes may be indicative of a shift toward a greater parasympathetically dominant autonomic profile, a state typically associated with enhanced cardiac stability and a lower risk of arrhythmias. However, as the HRV is an indirect method for sympathetic–parasympathetic balance measure, any inference regarding enhanced vagal activity or its potential stabilizing effect on cardiac electrophysiology remains speculative. Interestingly, these benefits appeared to stabilize after the initial 3 months, with no further significant changes observed over the subsequent 12 months.
- Stability of device electrical parameters: Throughout the study period, device parameters—including R-wave amplitude, ventricular pacing thresholds, and impedance—remained stable. This stability indicates that Sacubitril/Valsartan does not adversely affect the sensing or pacing functions of ICDs or CRT-Ds. This finding reinforces the safety of Sacubitril/Valsartan in patients with these devices.
- Impact on VA: The effect of Sacubitril/Valsartan on ventricular arrhythmic events was more nuanced. In the overall cohort, there was no statistically significant difference in VA incidence between the pre- and post-treatment periods. However, acknowledging that this result is hypothesis-generating and may be influenced by regression to the mean or other unmeasured factors, in the subgroup of patients with a history of arrhythmias before treatment initiation, a significant reduction in recurrent arrhythmic events was observed post-treatment. This suggests that Sacubitril/Valsartan may have a protective impact in high-risk patients with prior arrhythmic events, reducing the burden of recurrent arrhythmias.

### 4.1. Previous Studies

Several recent studies have investigated the effects of Sacubitril/Valsartan on autonomic function in patients with HFrEF, yielding mixed results. Some studies demonstrated an improvement in sympathetic autonomic activity [15,16,17] while others did not observe significant changes [18]. For instance, Giallauria et al. reported a significant improvement in heart rate recovery among 134 patients after one year of Sacubitril/Valsartan therapy [16]. In contrast, Pastor-Pérez et al. found no significant changes in autonomic function [18]. Boehmer et al. demonstrated a notable increase in parasympathetic tone after 3 months of therapy, suggesting that the observed improvements may be mediated by mechanisms such as volume unloading [12].

The heterogeneity in these findings may be attributed to several factors, including small sample sizes, differences in patient populations, and the influence of concomitant antiarrhythmic therapies like Amiodarone, which are known to affect HRV [19]. Additionally, most of these studies relied on 24 h HRV analysis using Holter ECG, which provides a snapshot of autonomic activity but is susceptible to daily fluctuations. Our study is the first to evaluate adrenergic tone using continuous data collected via implantable devices (ICDs and CRT-Ds). This method enabled us to analyze daily data, such as the standard deviation of the mean atrial interbeat interval in 5 min segments, averaged over a 7-day time window at multiple timepoints. By reducing the impact of autonomic tone variability, this approach allowed for a more accurate assessment of both short-term (3 months post-therapy) and long-term (12 months post-therapy) effects.

Furthermore, our results confirmed the stability of electrical parameters for the RV lead, including sensing amplitude, pacing thresholds, and impedance, throughout the study period. This finding is crucial for ensuring the safety and functionality of ICDs and CRT-Ds. Our results align with those of Russo et al., who reported similar stability in a cohort of 167 patients with dilated cardiomyopathy [20].

Unlike some previous studies, [4,20] we did not observe a statistically significant reduction in the overall burden of sustained VA. This may be due to the limited size of our cohort, which lacked the statistical power to detect such differences. However, in the subgroup of patients with a history of VA prior to therapy initiation, we observed a significant reduction in recurrent arrhythmic events post-therapy. Conversely, we also noted new-onset VA episodes after therapy initiation, as previously reported in the literature [21].

Available data do not allow for conclusions regarding a protective or adverse effect of Sacubitril/Valsartan on ventricular arrhythmia burden. Instead, they highlight the need for further investigation to clarify whether any relationship exists. Clinical decisions—such as temporarily adjusting therapy in the setting of new arrhythmic events—should therefore be made cautiously and individualized.

### 4.2. Clinical Implications

The findings of this study suggest that Sacubitril/Valsartan offers not only hemodynamic benefits for patients with HFrEF, but also contributes to favorable autonomic modulation. In patients with ICDs or CRT-Ds, particularly those with a history of cardiac arrhythmias, Sacubitril/Valsartan may provide additional antiarrhythmic benefits by reducing the frequency of ventricular arrhythmias. This effect is especially significant for managing patients with symptomatic HF who are prone to arrhythmias, as it could lower the frequency of defibrillator shocks. Such shocks can be distressing for patients and are associated with increased morbidity. Moreover, the observed improvement in HRV and reduction in mean and nocturnal heart rate emphasize Sacubitril/Valsartan’s role in addressing the autonomic imbalance frequently seen in HF. By enhancing parasympathetic activity, the drug helps to promote a more favorable autonomic profile. The availability in our study of patient activity, which showed no significant difference during time, allows us to rule out the possibility that the observed changes were related to sympathetic nerve activity triggered by increased physical stress. This modulation could have a meaningful impact on cardiovascular stability, reduce arrhythmic risk, and improve the overall quality of life for this patient population [22].

### 4.3. Limitations and Future Directions

This study has several limitations. The relatively small sample size, along with the inclusion of patients from a single country and healthcare setting, may limit the generalizability of the findings. Moreover, the limited sample size precludes subgroup analyses and multivariate adjustments. The retrospective nature of the data analysis also introduces a potential for bias.

While the therapy demonstrated significant benefits within the first three months, the lack of further improvements at 12 months raises questions about the long-term sustainability of these effects. This underscores the need for extended follow-up studies to determine whether the observed autonomic and antiarrhythmic benefits persist over time.

Future research should focus on larger-scale, prospective controlled studies to confirm these findings and provide more robust evidence. These studies should also aim to elucidate the mechanisms underlying the effects of Sacubitril/Valsartan on autonomic function and arrhythmia recurrence. Understanding these mechanisms could help identify patient subgroups most likely to benefit from therapy and optimize treatment strategies for managing HF and its associated complications.

The exclusion of patients with AF at baseline, a common comorbidity in HFrEF, limits the applicability of these findings to the broader HF population. In our analysis, we included only patients in sinus rhythm, due to technical constraints and to avoid the confounding effects of AF episodes on HRV. Nonetheless, future studies should specifically investigate patients with permanent or long-standing AF, stratifying them by AF type (e.g., paroxysmal, persistent, or permanent).

## 5. Conclusions

The analysis of physiological sensor data transmitted by implantable cardiac devices revealed that initiating Sacubitril/Valsartan therapy in patients with HFrEF was associated with small but statistically significant improvements in HRV and modest reductions in heart rate, reflecting a limited yet favorable autonomic modulation. Importantly, the drug did not affect the electrical parameters of the RV lead. Additionally, Sacubitril/Valsartan therapy significantly reduced the VA burden in patients with a history of arrhythmias prior to therapy. These findings support the use of Sacubitril/Valsartan in HFrEF patients with ICD or CRT-D.

## Figures and Tables

**Table 1:** Patient characteristics at device implantation.

Characteristic	Study Cohort (N = 54)
Age, years	68 (63–73)
Gender	
Male	46 (85%)
Female	8 (14%)
BMI, kg/m^2^	26.8 (23.5–29.4)
Device type	
ICD VR	1 (2%)
DX ICD	11 (20%)
ICD DR	15 (28%)
CRT-D	27 (50%)
NYHA class	
I	3 (8%)
II	30 (75%)
III	7 (17%)
SCD prevention	
Primary	45 (83%)
Secondary	9 (17%)
Cardiomyopathy	
Non-ischemic	22 (41%)
Ischemic	32 (59%)
LVEF, %	31.5 (28.0–35.0)
QRS duration, ms	108 (98–160)
Comorbidities	
Hypertension	32 (59%)
Dyslipidemia	25 (48%)
History of AF	15 (29%)
Diabetes	10 (19%)
COPD	5 (13%)
CKD	2 (5%)
Stroke/TIA	1 (3%)
Medical therapy	
ACEI/ARB	25 (54%)
Beta-blockers	43 (80%)
Anticoagulant	20 (50%)
Diuretic	43 (80%)
Amiodarone	8 (13%)

Values are reported as median (interquartile range) for continuous variables, and as count (percentages). Abbreviations: ACEI = angiotensin-converting enzyme inhibitor; AF = atrial fibrillation; ARB = angiotensin receptor blocker; BMI = body mass index; CKD = chronic kidney disease; COPD = chronic obstructive pulmonary disease; CRT-D = cardiac resynchronization therapy defibrillator; ICD = implantable cardioverter-defibrillator; ICD DR = dual-chamber ICD; DX ICD = single-lead ICD with atrial sensing dipole; ICD VR = single-chamber ICD; LVEF = left ventricular ejection fraction; NYHA = New York Heart Association; SCD = sudden cardiac death; TIA = transient ischemic attack.

**Table 2:** Physiological and device electrical parameters measured before Sacubitril/Valsartan treatment (pT), and at 3-month (3 mT) and 12-month (12 mT) follow-up after treatment initiation.

	Time of Assessment		
	pT	3 mT	12 mT
Physiological parameters			
HRV ^1^, ms	78.6 (54.2–104.6)	80.75 (60.8–108)	76.4 (61.4–108.0)
24 h-HR, bpm	73.2 (67.3–77.7)	69.9 (64.2–75.7)	71.9 (65.6–79.6)
nHR, bpm	63.0 (58.1–70.0)	60.4 (56.0–68.6)	62.6 (60.0–68.7)
PVC/h	2.7 (0–32.7)	1.9 (0–28.2)	1.9 (0–26.0)
CRT, %	99.3 (92.3–100)	99.2 (98.3–100)	99.2 (98.1–100)
DDAF burden, %	0.14 (0–41.3)	2.9 (0–44.3)	1.8 (0–47.7)
Patient activity, %	10.0 (7.0–15.3)	10.5 (7–13.4)	10.6 (6–17.1)
Device electrical parameters			
R wave amplitude, mV	14.8 (8.9–19.1)	15.5 (9.12–19.4)	13.9 (6.3–19.9)
RV pacing threshold @ 0.4 ms, V	0.7 (0.6–1.0)	0.7 (0.6–0.9)	0.7 (0.5–0.9)
RV pacing impedance, Ohm	475 (442–535)	489 (461–529)	495 (473–521)
Shock impedance, Ohm	81 (64–86)	79 (67–86)	77 (70–86)

Data are reported as median (interquartile range). ^1^ HRV could not be calculated for the patient with a single-chamber ICD and for those with atrial fibrillation during the analyzed period. Abbreviations: 12 mT = 12 months after treatment initiation; 24 h-HR = mean heart rate over 24 h; 3 mT = 3 months after treatment initiation; bpm = beats per minute; CRT = cardiac resynchronization therapy; DDAF = device-detected atrial fibrillation; HRV = heart rate variability; nHR = nocturnal heart rate; pT = pre-treatment; PVC/h = premature ventricular contractions per hour; RV = right ventricular.

**Table 3:** Mixed models linear regression analysis of variations in physiological and device electrical parameters across the three time points: before Sacubitril/Valsartan treatment (pT), at 3-month (3 mT), and 12-month (12 mT) follow-up after treatment initiation.

	Time Comparison	Regression Coefficient	Standard Error	*p*-Value
Physiological parameters				
HRV	pT–3 mT	5.58	2.73	0.041
	3 mT–12 mT	0.36	1.58	0.821
24 h-HR	pT–3 mT	−2.49	1.03	0.016
	3 mT–12 mT	0.04	0.51	0.932
nHR	pT–3 mT	−2.28	1.03	0.028
	3 mT–12 mT	0.19	0.51	0.710
PVC/h	pT–3 mT	3.08	11.0	0.779
	3 mT–12 mT	−5.01	4.12	0.225
DDAF burden, %	pT–3 mT	−0.72	2.49	0.773
	3 mT–12 mT	−0.56	0.45	0.217
CRT, %	pT–3 mT	1.91	1.04	0.066
	3 mT–12 mT	0.25	0.23	0.281
Patient activity, %	pT–3 mT	−0.35	0.55	0.521
	3 mT–12 mT	−0.39	0.50	0.429
Device electrical parameters				
R wave amplitude	pT–3 mT	0.03	0.30	0.927
	3 mT–12 mT	−0.32	0.27	0.247
RV pacing threshold	pT–3 mT	0.01	0.00	0.561
	3 mT–12 mT	−0.01	0.01	0.237
RV pacing impedance	pT–3 mT	3.82	3.83	0.319
	3 mT–12 mT	−3.33	3.80	0.381
Shock impedance	pT–3 mT	0.42	0.82	0.606
	3 mT–12 mT	−0.04	0.51	0.936

Abbreviations: 12 mT = 12 months after treatment initiation; 24 h-HR = mean heart rate over 24 h; 3 mT = 3 months after treatment initiation; CRT = cardiac resynchronization therapy; DDAF = device-detected atrial fibrillation; HRV = heart rate variability; nHR = nocturnal heart rate; pT = pre-treatment; PVC/h = premature ventricular contractions per hour; RV = right ventricular.

## Data Availability

The data underlying this article will be shared on reasonable request to the corresponding author.

## Abbreviations

The following abbreviations are used in this manuscript:

AF	Atrial fibrillation
CRT-D	Cardiac resynchronization therapy defibrillator
CRT%	Percentage of cardiac resynchronization therapy pacing
DDAF	Device-detected atrial fibrillation
HF	Heart failure
HFrEF	HF with reduced ejection fraction
HRV	Heart rate variability
ICD	Implantable cardioverter-defibrillator
nHR	Nocturnal heart rate
PVC/h	Frequency of premature ventricular contractions per hour
VA	Ventricular arrhythmia
24 h-HR	Mean heart rate over 24 h
